# Calcium imaging of adult-born neurons in freely moving mice

**DOI:** 10.1016/j.xpro.2020.100238

**Published:** 2020-12-31

**Authors:** Alvaro Carrier-Ruiz, Yuki Sugaya, Deependra Kumar, Pablo Vergara, Iyo Koyanagi, Sakthivel Srinivasan, Toshie Naoi, Masanobu Kano, Masanori Sakaguchi

**Affiliations:** 1 Department of Neurophysiology, Graduate School of Medicine, The University of Tokyo, Tokyo 113-0033, Japan; 2 International Research Center for Neurointelligence (WPI-IRCN), The University of Tokyo Institutes for Advanced Study (UTIAS), Tokyo 113-0033, Japan; 3 International Institute for Integrative Sleep Medicine (WPI-IIIS), University of Tsukuba, Tsukuba, Ibaraki 305-0006, Japan; 4 Department of Neuroscience, Karolinska Institute, Stockholm 17165, Sweden

**Keywords:** Microscopy, Model Organisms, Neuroscience

## Abstract

Adult-born neurons (ABNs) in the dentate gyrus bestow unique cellular plasticity to the mammalian brain. We recently found that the activity of ABNs during sleep is necessary for memory consolidation. Here, we describe our method for Ca^2+^ imaging of ABN activity using a miniaturized fluorescent microscope and sleep recordings. As preparatory surgery and post-recording data processing can be major obstacles, we provide detailed descriptions and problem-solving tips.

For complete details on the use and execution of this protocol, please refer to Kumar et al. (2020).

## Highlights

• Express a Ca^2+^ indicator, GCaMP3, to label adult-born neurons
• Perform surgery, Ca^2+^ imaging, sleep recording, and fear conditioning
• Process data to track the same adult-born neurons across lengthy recording sessions

## Graphical Abstract

**Figure:**
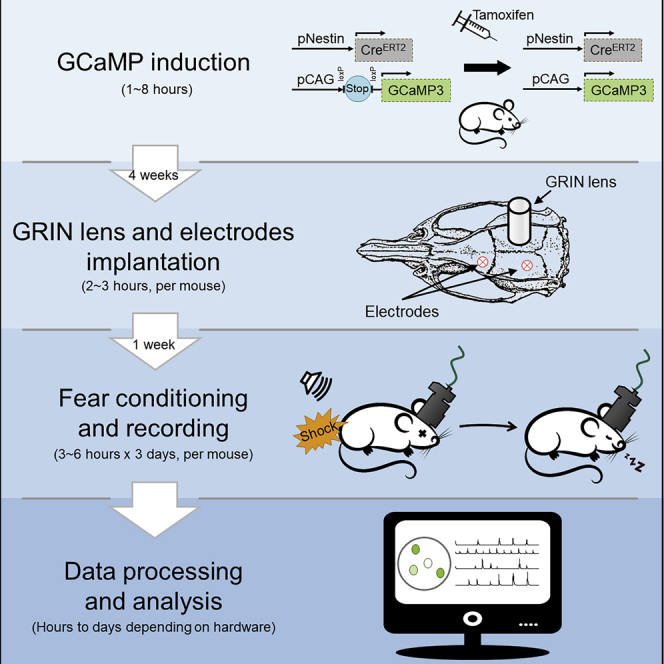

## Before you begin

All experiments were performed in accordance with the Science Council of Japan’s Guidelines for Proper Conduct of Animal Experiments. Experimental protocols were approved by the Institutional Animal Care and Use Committee at the University of Tsukuba. C57BL/6 mice (Jackson Laboratory) were bred in our colony at the University of Tsukuba and maintained on a 12-h light/dark cycle (lights on 9 am to 9 pm) with ad libitum access to food and water. All mice were group-housed with 2–5 mice/cage, and only male mice were used in the experiments. To induce transgene expression in ABNs, mice were treated with tamoxifen at 7 weeks of age. At ∼9–12 weeks of age, mice were anesthetized with isoflurane and fixed in a stereotaxic frame for the surgery demonstrated in the videos.

### Generate transgenic mice

**Timing: 3–4 months**

1. Obtain at least two transgenic mouse lines: one that expresses tamoxifen-inducible Cre recombinase in neural stem/progenitor cells, and one that expresses a fluorescent calcium (Ca^2+^) indicator protein after exposure to Cre recombinase.

***Alternatives:*** We successfully used Nestin-CreERT2 transgenic mice from The Jackson Laboratory (stock #016261), in which Cre recombinase is expressed in Nestin-positive cells in the adult brain by tamoxifen induction. Alternatively, Ascl1-CreERT2 mice (The Jackson Laboratory, stock #012882) could be used to specifically target transient amplifying progenitors (Kim et al., 2007, Yang et al., 2015), although we do not have experience using this line.

***Note:*** We recommend using the GCaMP3 mouse line Ai38 (RCL-GCaMP3) to express the fluorescent Ca^2+^ indicator in adult-born neurons (ABNs). Although next-generation indicators, such as GCaMP6f and GCaMP6s, are usually preferable, their mouse lines (Madisen et al., 2015) show very low or absent expression of Cre-inducible transgene when expressed in ABNs after crossing with Nestin-CreERT2 mice. For example, crossing Nestin-CreERT2 mice with Ai96 (RCL-GCaMP6s) or Ai94 (TRE-LSL-GCaMP6s)/RCL-tTA mice (The Jackson Laboratory, stock #008600) does not induce expression of GCaMP6 (unpublished results). We speculate that expression of recently developed GCaMP sensors in early neural progenitors interferes with Ca^2+^ signaling mechanisms essential for immature ABN survival.

***Note:*** Instead of using mouse transgenic lines, induction of Ca^2+^ indicators and/or Cre recombinase could be achieved by viral transduction. However, most adeno-associated viruses (AAVs) are not suitable for infecting neural stem/progenitor cells due to their low infection rate (Kotterman et al., 2015) and potential cytotoxicity (Johnston et al., 2020). Although AAV-mediated expression of GCaMP6 was reported in combination with two AAVs (Danielson et al., 2016), this previous study did not confirm expression of GCaMP6 in progenitor cells.

2. Cross transgenic lines, in accordance with their corresponding guidelines, to generate mice in which expression of GCaMP3 is initiated by tamoxifen-inducible Cre recombinase (i.e., CreERT2) in adult neural stem/progenitor cells (i.e., nestin:GCaMP3 mice).

## Key resources table

**Table:** 

REAGENT or RESOURCE	SOURCE	IDENTIFIER
**Chemicals, peptides, and recombinant proteins**		

Tamoxifen	Merck	T5648-1G
Isoflurane inhalation solution	Pfizer	n/a
Ibuprofen	Wako, Japan	094-02643
Xylocaine injection 1% with epinephrine (lidocaine)	Aspen Japan	n/a
OCT mounting medium	Sakura Finetek	4583
Vectashield	Vector	H-1000
4% paraformaldehyde phosphate buffer solution	Nacalai Tesque	09154-85
Sucrose	Nacalai Tesque	30404-45
Sunflower oil	Sigma	1642347
Corn oil	Sigma	C8267
Flunixin meglumine	Fujita	FLUNIXIN INJ. 10%
Antibiotics (sulfadiazine/trimethoprim)	Kyoritsu Seiyaku	Tribrissen injection
Tarivid ophthalmic ointment 0.3% (Ofloxacin)	Santen Pharmaceutical	1319722M1056

**Experimental models: organisms/strains**		

Mice: C57BL/6-Tg(Nes-cre/ERT2)KEisc/J	The Jackson Laboratory	016261
Mice: Ai38(RCL-GCaMP3)	The Jackson Laboratory	029043

**Software and algorithms**		

nVista HD	Inscopix	n/a
Inscopix image decompressor	Inscopix	n/a
EEG and EMG recording and analysis software	KISSEI COMTEC	Vital Recorder and Sleep Sign
Mosaic	Inscopix	n/a
MATLAB	MathWorks	2016b
IgorPro 8	WaveMetrics	n/a

**Other**		

∗GRIN lens probe, 1.0-mm diameter, ~4.0-mm length	Inscopix	1050-004595
∗Rectangular metal frame	Narishige	CF-10
∗Baseplate	Inscopix	1050-004638
∗Miniscope	Inscopix	nVista(2.0)
∗Baseplate cover	Inscopix	1050-004639
∗Miniscope gripper	Inscopix	1050-002199
∗Dummy miniscope	Inscopix	1050-003762
∗∗Electrodes for EEG recording	Yamazaki	ϕ1.0 × 2.0
∗∗Electrodes for EMG recording	Cooner Wire	AS633
∗∗Flat cable for EEG/EMG signal transfer	Hitachi Cable	20528-ST LF
∗∗EEG/EMG signal amplifier	Kyoto Biotex	n/a
∗∗Analog-to-digital convertor for EEG/EMG signal	Contec	AD16-16U(PCIEV)
∗∗4-pin header for EEG/EMG socket	Hirose	A3B-4PA-2DSA(71)
Fear conditioning setup	Med Associates	n/a
Isoflurane vaporizer	Shinano Seisakusyo	SN-487-OT
Stereotaxic frame	Narishige	SR-6M
Drill	Minitor	Minimo
Vacuum pump	Iwaki	VPUMP-140
Surgical microscope	Leica	Leica GZ6
Dental cement liquid	Shofu	PROVINICE 204310644
Dental cement powder	Shofu	PROVINICE 096017656
Loctite cyanoacrylate adhesive	Loctite	Loctite 454
Super glue	Konishi	Alon Alfa A
Silicone	Shofu	DENTSILICONE-V
Head-fixing support	Narfishige	MAG-1
Perista perfusion pump	ATTO	AC2110
Cryostat	Leica Microsystems	Leica CM 1850
Confocal fluorescent microscope	Olympus	FV1200
Glass electrodes	Harvard Apparatus	GC150F-7.5
Ultrasonic bath	SND	US-101
Glass electrode puller	Narishige	PC-10

**Deposited data**		

Data and code underlying the results described in this manuscript	https://github.com/vergaloy/ABNs-Calcium-extraction	n/a
Raw data from the related article (Kumar et al., 2020)	https://data.mendeley.com/datasets/gfbdv5kfrz/1	n/a

∗Materials only for imaging surgery.

∗∗Materials only for EEG/EMG surgery.

## Step-by-step method details

### Induction of Ca^2+^ sensor expression in ABNs

**Timing: 1–8 h**

Express GCaMP3 via tamoxifen-inducible Cre recombinase in adult neural stem/progenitor cells.

1. Prepare 20 mg/mL tamoxifen solution.
  a. Heat corn oil to 42°C for 30 min.
  b. Dissolve tamoxifen in corn oil at a concentration of 20 mg/mL in an ultrasonic bath (generic consumer glassware is sufficient) or with continuous shaking at 37°C. Store the prepared solution at 4°C.

**CRITICAL:** As tamoxifen is sensitive to light, it must be made and stored away from light in an amber or foil-wrapped tube.

***Note:*** Tamoxifen may take 1–7 h to completely dissolve depending on the dissolution method.

2. Inject tamoxifen into nestin:GCaMP3 mice.
  a. Select an appropriate number of adult nestin:GCaMP3 mice.
    ***Note:*** The definition of “adult” is usually determined by sexual maturity, which is commonly accepted to be ∼6 weeks of age for mice. Older or younger mice can be used if needed. However, older mice have lower levels of adult neurogenesis, which decreases the success rate of imaging, whereas younger mice might be too delicate to survive the necessary surgical procedures.
  b. Administer tamoxifen (120 mg/kg) into the intraperitoneal cavity five times at 1- or 2-day intervals. Store the solution at 4°C until the next injection.

**CRITICAL:** Avoid using tamoxifen that has been stored for more than 1 week. The efficiency of tamoxifen-induced recombination is severely impaired when using old or light-exposed tamoxifen.

***Note:*** The duration of the injection period is correlated with the degree of developmental variation of labeled ABNs. That is, the longer the injection period, the higher the chance of recording ABNs with different properties resulting from differences in their maturational stages.

### Maturation of labeled ABNs

**Timing: dependent on experimental design**

Allow ABNs to mature after tamoxifen injection.

3. Wait a pre-determined period of time to allow GCaMP3-labeled ABNs to mature.

***Note:*** The length of the waiting period depends on the target age of ABNs at the time of Ca^2+^ imaging. Keep in mind that 1–2 weeks is needed to prepare for imaging. For example, if the target age of ABNs is 6 weeks, wait 4 weeks after tamoxifen injection to move on to the next step. If the target age of ABNs is 1 week or less, consider performing lens implantation before tamoxifen injection. So far, we have successfully performed Ca^2+^ imaging of ABNs as early as 3 days after the last tamoxifen injection.

**Figure 1:**
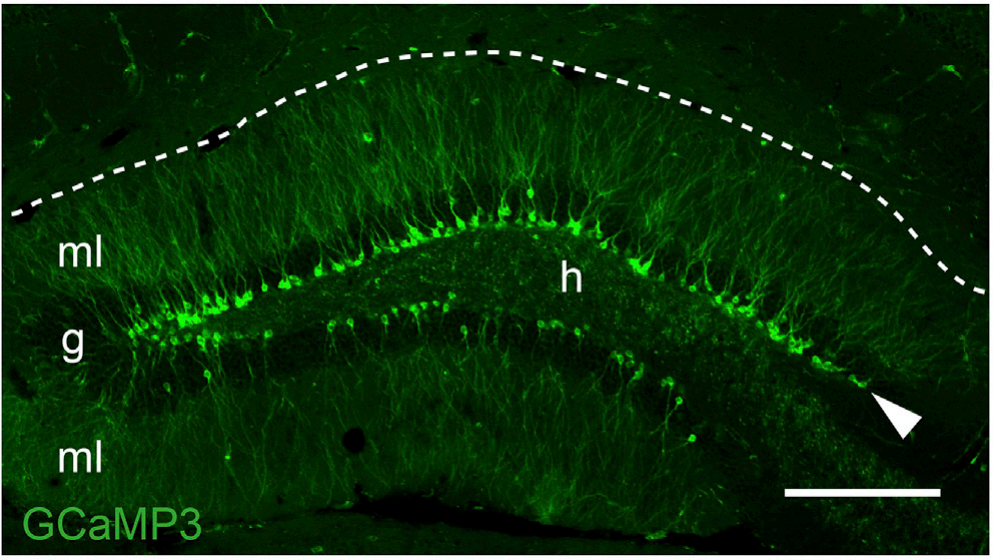
Adult-born neurons (ABNs) expressing the calcium (Ca^2+^) sensor GCaMP3 Optical section of the dentate gyrus (DG) with confocal microscopy showing ABNs expressing the Ca^2+^ sensor GCaMP3 induced by tamoxifen injection. The letters g, h, and ml represent the granule cell layer, hilus, and molecular layer, respectively. The arrowhead indicates the subgranular zone, and the dotted line indicates the border between the DG and cornu ammonis 1 (CA1). Scale bar, 100 μm.

***Note:*** GCaMP3 expression can be verified by examining brain sections from a tamoxifen-injected mouse using a fluorescence microscope (Figure 1) before the next step.

### GRIN lens and EEG/EMG electrode implantation

**Timing: 2–3 h per mouse**

Surgically implant a gradient-index (GRIN) lens and electroencephalogram (EEG)/electromyogram (EMG) electrodes into the brains of tamoxifen-injected mice.

***Note:*** We describe a specific protocol for imaging ABNs in the dentate gyrus (DG) in freely moving mice. The original miniscope technology used in this protocol was first developed by Ghosh et al., (2011). For a more generalized protocol to image other brain regions, please refer to Resendez et al. (2016) for a recent overview. *In vivo* Ca^2+^ imaging of ABNs in the DG was first described by Danielson et al. (2016) in awake head-fixed mice.

***Note:*** We implanted EEG/EMG electrodes to identify sleep stages in combination with ABNs activity. Mammalian sleep contains two major orthogonal states: rapid eye movement (REM) sleep and non-REM (NREM) sleep. Each sleep stage is characterized by prominent neural oscillatory patterns. For example, slow waves in the cortex are abundant during deep NREM sleep, whereas theta oscillations in the hippocampus occur during REM sleep, both of which play critical roles in memory consolidation (Boyce et al., 2016; Marshall et al., 2006). Details of sleep recording and analysis are available in Oishi et al. (2016).

**Figure 2:**
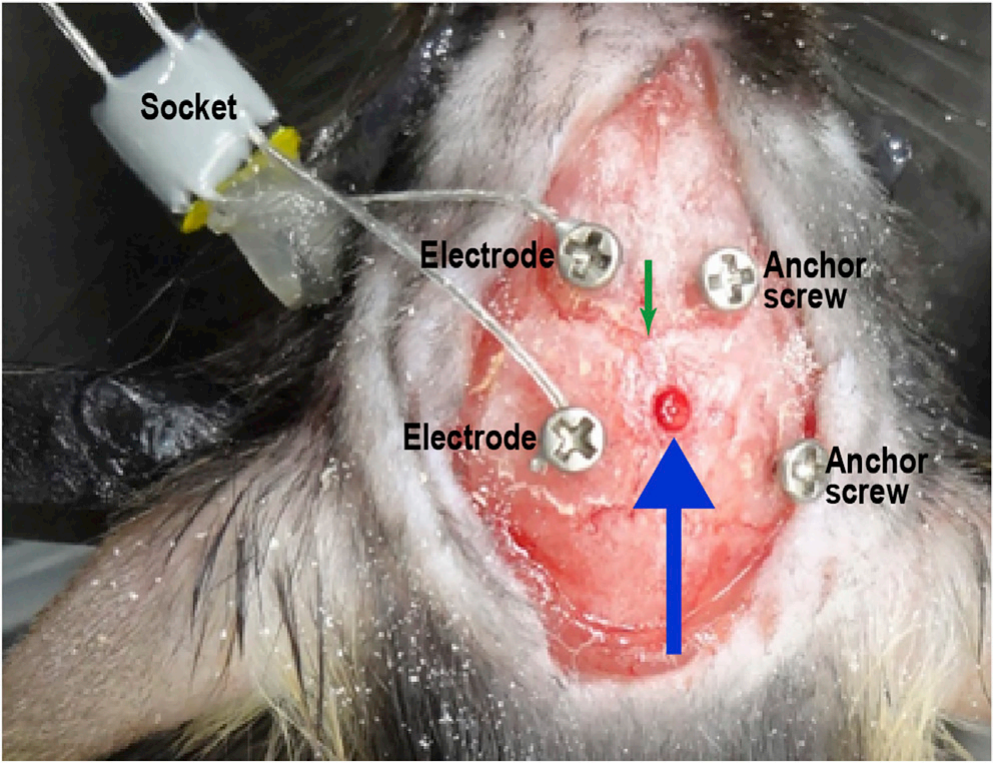
Positions of the four screws and hole for the gradient-index (GRIN) lens The blue arrow indicates the GRIN lens position. The green arrowhead indicates bregma.
    ***Note:*** The electrode holes should be just large enough to accommodate the tip of the electrodes. In Figure 2, the holes were drilled above the frontal and parietal cortices on both sides of skull as an example: anteroposterior (AP) +1.5 mm and −3 mm and mediolateral (ML) ±1.5 mm and ±1.7 mm, respectively. The anchoring electrodes prevent the implant from becoming detached at later points in the experimental protocol.
    Methods Video S1. Drilling holes for implanting the GRIN lens and EEG electrodes, refer to steps 5j and k and 6a
  k. Make a cranial window for GRIN lens implantation using a drill (Methods Video S1, 00:30–00:41).
    ***Note:*** The cranial window should be just large enough to accommodate the GRIN lens implant. We recommend using an Inscopix GRIN lens 1 mm in diameter and 4 mm in length to record ABNs in the dorsal DG. In this case, a 1.2-mm^2^ cranial window centered at AP −2 mm and ML +0.7 mm works well.

**Figure 3:**
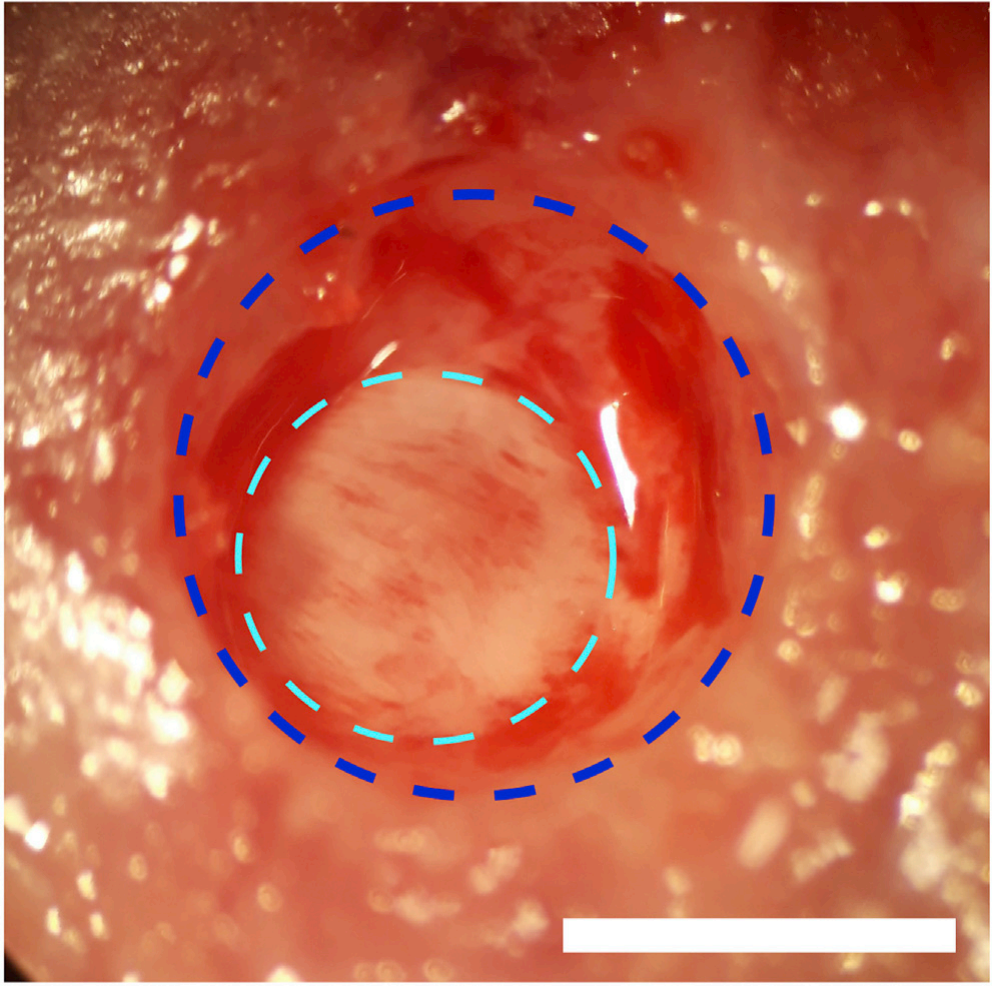
White matter tracts with white striations exposed during aspiration of cortical tissue The area of white matter tracts is surrounded by the light blue dotted line. The cranial window (blue dotted line) is larger than the exposed area. Scale bar, 1 mm.
    ***Note:*** From this point onward, some bleeding is normal during the procedure. Continuously irrigate the exposed brain tissue with sterile saline while aspirating the cortex (Methods Video S2, 00:13–00:23).
    **CRITICAL:** A surgical microscope is needed to observe distinctions in brain structure during surgery. In our experience, damaging the CA1 by aspiration usually destroys the structural organization of the DG.
    Methods Video S2. Aspirating cortical tissue for GRIN lens implantation, refer to step 6b and c
  c. After reaching the corpus callosum fibers, keep the exposed brain tissue irrigated with sterile saline while aspirating any blood present in the region to maintain visibility (Methods Video S2, 00:37–00:53).
  d. Attach a GRIN lens to a stereotaxic manipulator using a clip holder (Figure 4) and align it above the center of the cranial window (Methods Video S3, 00:00–00:15).

4. Anesthetize the mouse with an anesthetic of choice (e.g., isoflurane).

5. Open a cranial window sufficiently large for lens and electrode implantation.
  a. Shave hair from the surgical site.
  b. Secure the head of the mouse in a stereotaxic frame.
  c. Cover the eyes of the mouse with white petrolatum cream to prevent dryness.
  d. Disinfect the skin at the surgical site.
  e. Make a small incision in the skin using a spring scissor along the sagittal line of the skull.
  f. Clean the exposed skull with cotton swabs.
  g. Scratch the skull surface using a drill bit to make it rough, which will facilitate later adhesion of glue and dental cement.
  h. Obtain a flat skull position by matching the height of two parallel points along the sagittal axis (±3 mm lateral from bregma) as well as the height of bregma and lambda points.
  i. Mark the desired stereotaxic coordinates for the GRIN lens and EEG electrodes on the skull surface using the stereotaxic manipulator.
  j. Drill five holes in the skull: one for the GRIN lens, two over one hemisphere for EEG electrodes, and two over the contralateral hemisphere for anchoring electrodes (i.e., not attached to EEG wires; Figure 2, Methods Video S1, 00:00–00:30).

6. Implant the EEG electrodes, anchoring screws, and GRIN lens.
  a. Insert the EEG electrodes and anchoring screws epidurally (i.e., not completely penetrating the skull; Figure 2, Methods Video S1, 00:42–01:31).
  b. For the lens hole, aspirate cortical tissue above the region of interest using a glass pipette connected to a vacuum pump in circular movements from the center to the periphery of the cranial window (Methods Video S2, 00:00–00:36). While aspirating the cortex, the brain tissue will first have a homogeneous appearance. Stop aspiration when white matter tracts of the corpus callosum and alveus hippocampus are visible as identified by striations (Figure 3).

**Figure 4:**
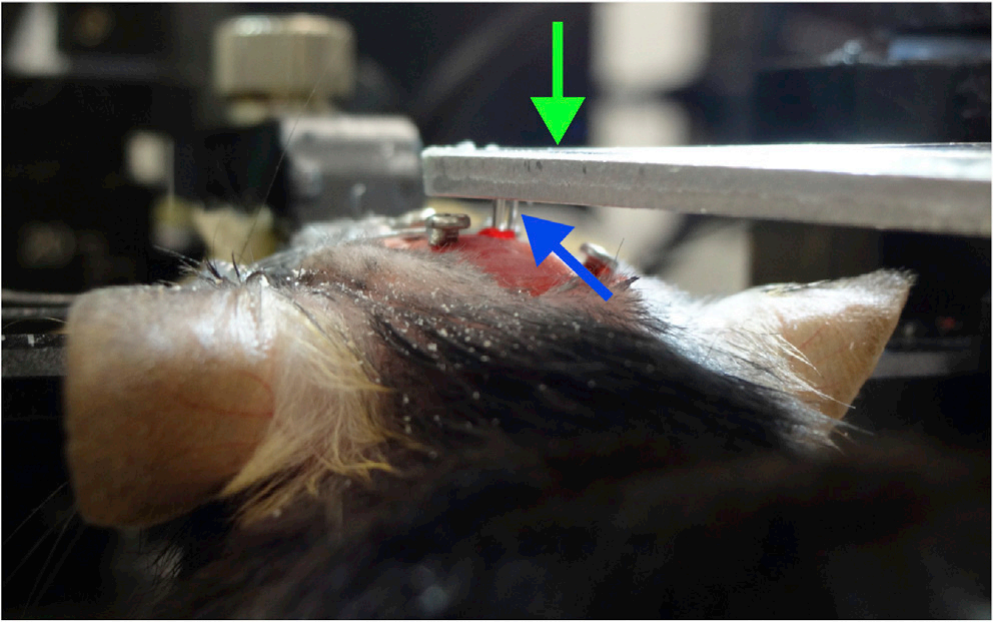
Setup for GRIN lens insertion A GRIN lens (blue arrow) is held by a clip holder (green arrow) and inserted into the hippocampus.
    ***Note:*** We made a lens holder by gluing a generic spring clip holder to the base of a manipulator bar. We recommend holding the GRIN lens at the tip (∼0.5-mm length) of the clip holder and keeping the lens straight (Methods Video S3, 00:00–00:09).
    Methods Video S3. Implanting the GRIN lens, refer to step 6d–h
  e. Carefully lower the lens using 0.1-mm dorso-ventral steps into the hippocampus. The final coordinate of the objective surface of the GRIN lens is 1.3 mm lower than the top of the skull (Methods Video S3, 00:16–00:34).
    ***Note:*** We lower the lens at a rate of ∼500 μm/min. Tissue swelling and relaxation affect the quality of subsequent recordings. We do not recommend using a smaller diameter lens because the resulting reduction in field of view makes it substantially more difficult to observe a population of ABNs that is sparse in both number and activity.
  f. Fix the GRIN lens to the skull and all four screws with a thin layer of cyanoacrylate adhesive (Loctite 454, referred to henceforth as Loctite glue) and allow it to cure for ∼5–10 min (Methods Video S3, 00:35–00:53).
    ***Note:*** We find that adding dental cement liquid on top of Loctite glue rapidly solidifies the adhesive and reduces this step to a few minutes.
  g. Release the lens from the stereotaxic manipulator (Methods Video S3, 00:54–01:00).
  h. Cover the skull using a layer of Loctite glue and dental cement liquid around the insertion point (Methods Video S3, 01:00–01:23) and allow it to completely cure.
  i. Attach the EEG/EMG socket to the skull using Loctite glue, add a drop of dental cement liquid, and allow it to completely cure (Methods Video S4, 00:00–00:11).
    **CRITICAL:** The EEG/EMG socket should be fixed far enough from the lens to avoid later collision between the socket and microscope.
    Methods Video S4. Implanting EEG/EMG electrodes, refer to step 6i–7
  j. Insert two wires into the cervical portion of the trapezoid muscles for EMG recording (Methods Video S4, 00:12–00:33).
  k. Apply carbon black powder-mixed dental cement (liquid + powder) to any exposed area of the skull as well as to the area where the Loctite glue meets the GRIN lens and EEG/EMG wires and let it solidify for ∼10 min (Methods Video S4, 00:34–00:49).
    **CRITICAL:** Completely cover the EEG/EMG wires with Loctite glue to prevent damage when the mouse scratches its head (Figure 5).
    **Figure 5:**
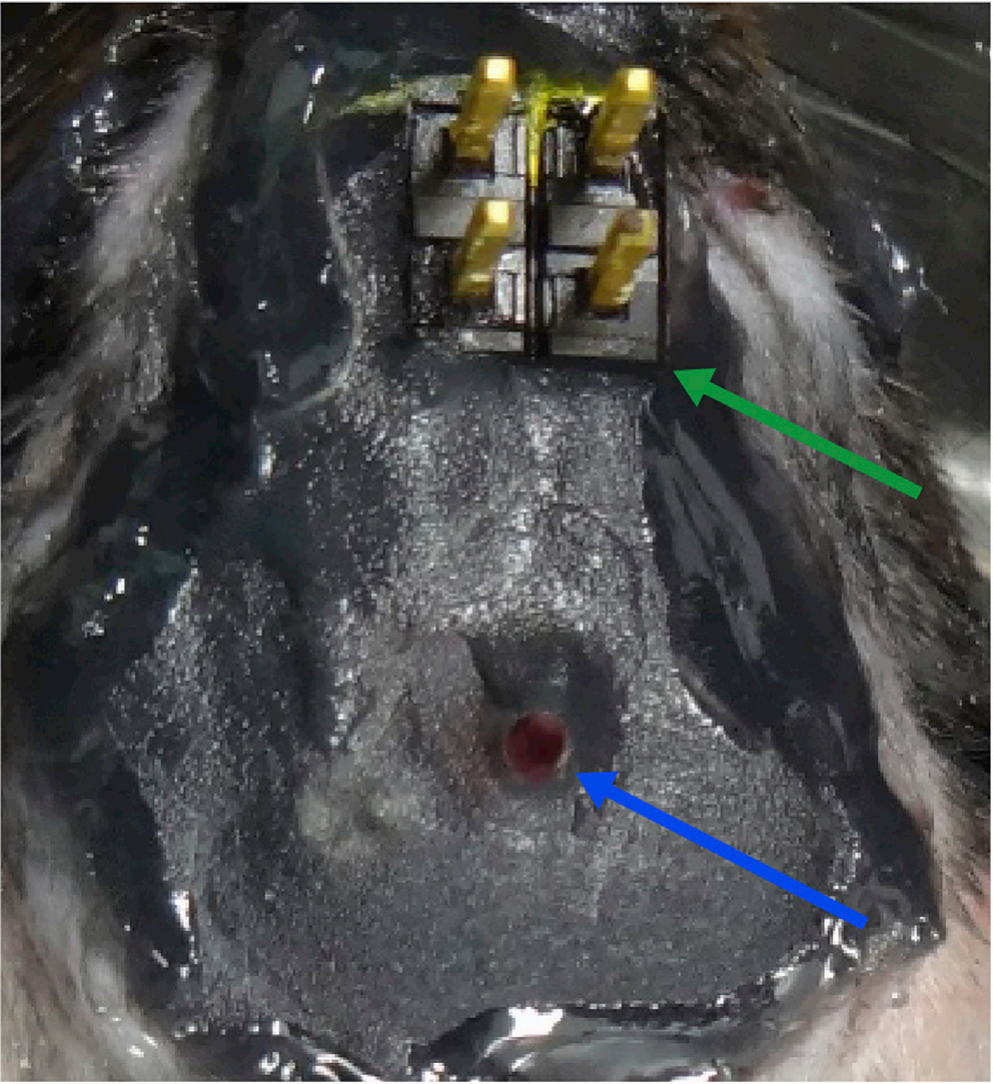
Electrodes and GRIN lens fixed with dental cement The skull and electroencephalogram (EEG)/electromyogram (EMG) wires are completely covered with black dental cement. Note that the EEG/EMG socket (green arrow) and GRIN lens (blue arrow) are not covered.
  l. Apply Loctite glue to the outer border of the dental cement to connect it to the surrounding skin and wait until it completely cures (Methods Video S4, 00:50–01:00).

**Figure 6:**
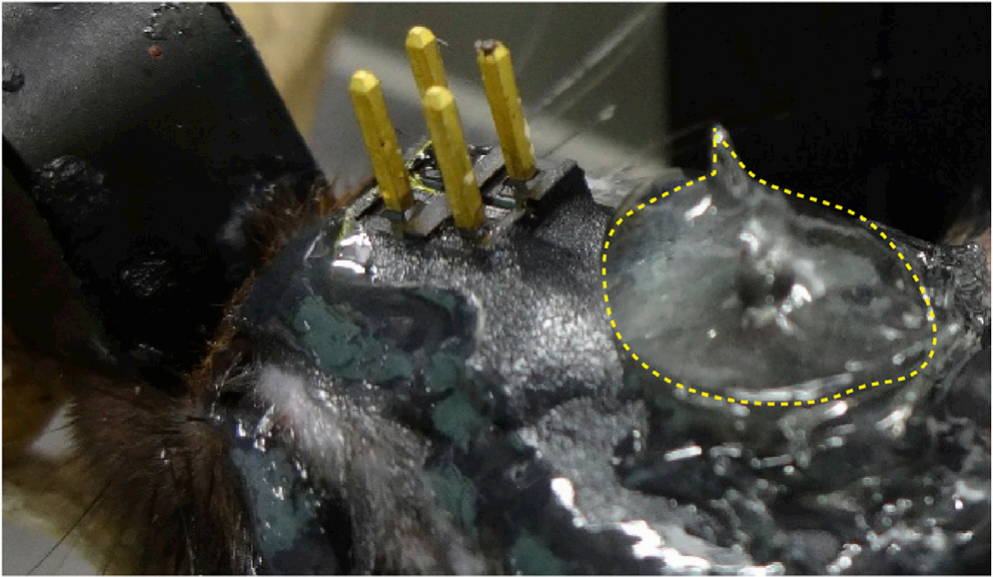
GRIN lens and the surrounding area covered with silicone The silicone is indicated by dotted line.

7. Cover the GRIN lens and surrounding area with silicone to protect it from damage resulting from mouse activity in the home cage (Figure 6, Methods Video S4, 01:01–01:12).

***Optional:*** Attach a rectangular metal frame (12 × 19 × 1 mm) to the skull using dental cement and let it solidify for 5 min. This frame will prevent the mouse from scratching the lens and aid in manipulating the awake mouse when attaching the miniaturized fluorescent microscope (miniscope) and EEG/EMG cables before recording.

8. Finish the surgical procedure.
  a. Stop anesthesia.
  b. Remove the mouse from the stereotaxic holder.
  c. Administer postoperative 5% glucose solution and ibuprofen (30 mg/kg).
  d. Place the mouse in a clean cage partially covering a heating pad to prevent hypothermia and allow it to recover.
9. House the operated mouse individually to avoid damage to the implant.

***Note:*** To prepare ibuprofen (30 mg/kg) solution, dissolve 30 mg ibuprofen powder in 100 μL of 100% ethanol, add 1 mL sunflower oil, and evaporate the ethanol by centrifuging.

### Postoperative recovery

**Timing: at least 1 week**

Allow operated mice to rest for at least 1 week with minimal manipulation.

***Note:*** We recommend keeping mice in the sleeping chamber/room during recovery to habituate them to the environment.

***Note:*** Manipulation of mice during this period may cause detachment of the lens and electrodes, as some degree of inflammation might still be present, making the implant more vulnerable to detachment from the skull.

### Miniscope baseplate attachment

**Timing: 30 min per mouse**

Attach a magnetic baseplate for the miniscope to lens-implanted mice.

**Figure 7:**
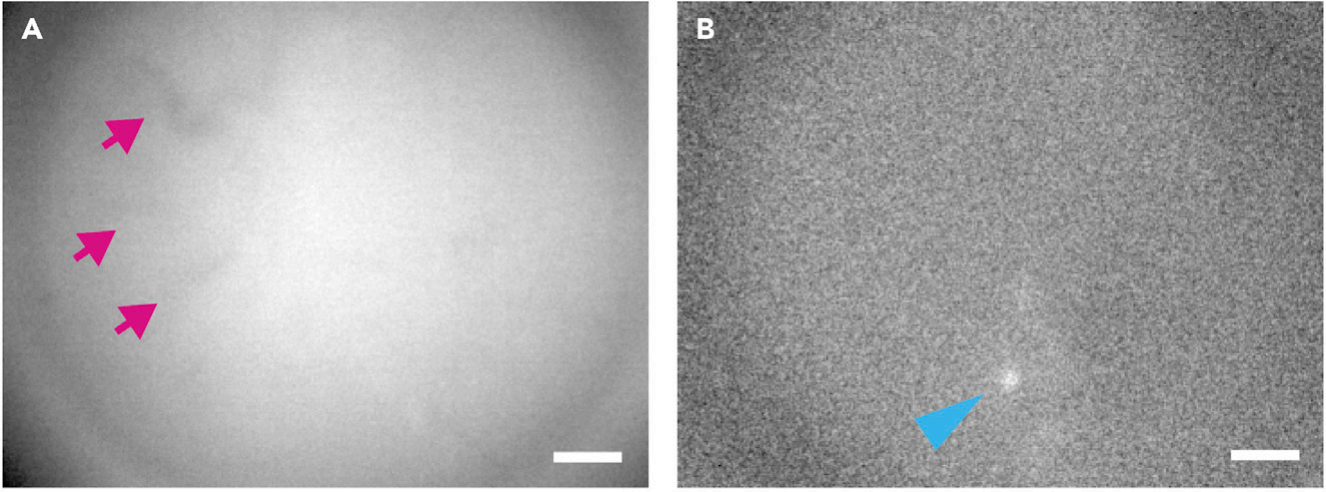
Fluorescent signals from the DG observed through the GRIN lens The DG is shown (A) without or (B) with ΔF/F processing. Red arrows indicate vasculature, and the blue arrowhead indicates a Ca^2+^ transient in an ABN. Scale bar, 100 μm.
  f. After choosing the optimal focal plane, secure the baseplate to the skull using dental cement (liquid + power) and allow it to completely cure (Figure 8, Methods Video S5).

10. Anesthetize the mouse with an anesthetic of choice (e.g., isoflurane).
11. Secure the head of the mouse in the stereotaxic frame.
12. Prepare the miniscope.
  a. Attach the magnetic baseplate to the miniscope.
  b. Secure the miniscope to its gripper tool and attach it to a micromanipulator.
  c. Connect the recording hardware to the computer.
  d. Position the miniscope above the head of the mouse.
  e. Start the image acquisition software.

13. Attach the magnetic baseplate.
  a. Remove the silicone protection from the skull of the mouse.
  b. Adjust the position of the miniscope using the micromanipulator, centering it above the implanted lens.
  c. Turn on the LED light of the miniscope and lower it until the top surface of the lens becomes visible through the camera.
  d. Carefully continue lowering the miniscope until the DG becomes visible.
    ***Note:*** The DG will be recognizable by its abundant vasculature. Due to GCaMP3 expression in ABNs and our implantation method, this will be the first focusable structure.
  e. From this point, to adjust the imaging focus to the granular/subgranular layer, lower the miniscope ∼50 μm. The vasculature should become slightly out of focus at this point (Figure 7A).
    ***Optional:*** When the outlines of GCaMP3-expressing ABNs can be visualized, we recommend switching to the nVista software ΔF/F function to confirm the presence of dynamic changes in fluorescence corresponding to Ca^2+^ transients (Figure 7B).
    ***Note:*** Due to the low signal-to-noise ratio of GCaMP3 and the small number of ABNs labeled using our protocol, the fluorescence signal from ABNs may be undetectable at this point. It may still be possible to observe ABN activity with post-recording processing of the images. The success rate is ∼50%.

**Figure 8:**
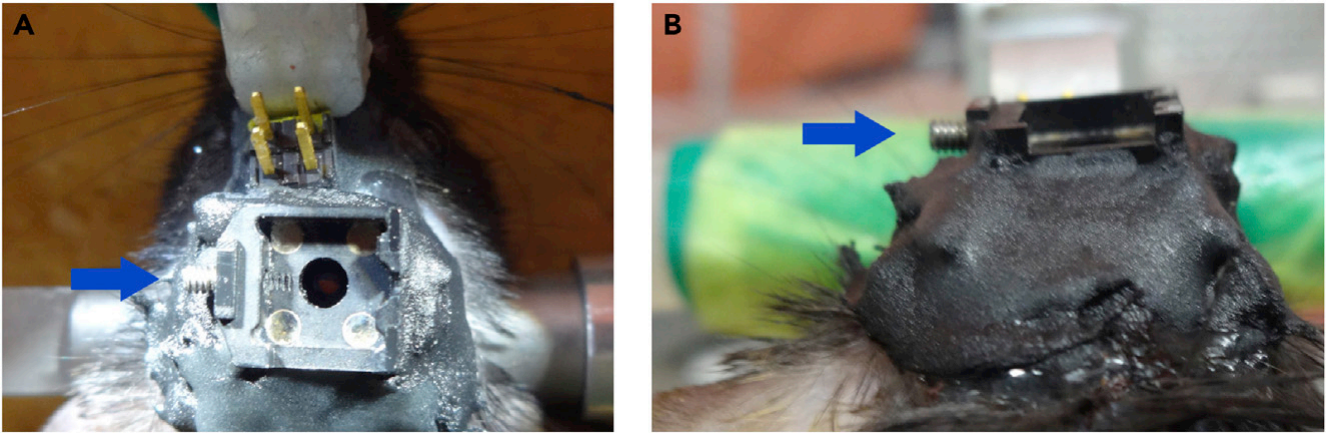
Black baseplate secured above the GRIN lens using black dental cement (A) Top view. (B) Rear view. Note that the screw (blue arrows) and the surface of the baseplate are not covered by dental cement.
    **CRITICAL:** When fixing the baseplate, there are three points at which dental cement should not be directly applied: the surface of the implanted lens, the screw for the magnetic baseplate, and the miniscope.
    ***Note:*** Fixing the baseplate without gaps will prevent dust and ambient illumination from interfering with the field of view during imaging.
    ***Note:*** Generic dental cement may shrink to a small degree when it becomes solid. If the position of the baseplate changes significantly, setting the baseplate 5–10 μm above the optimal focal plane before applying dental cement or using C&B Metabond as recommended by Inscopix may be helpful. In addition, the focal plane can be adjusted at the time of recording by turning the microscope as instructed by Inscopix.
    Methods Video S5. Fixing the baseplate, refer to step 13f

14. Finish the imaging preparation.
  a. After the dental cement has solidified completely, release the miniscope from the gripper tool.
  b. Detach the miniscope from the magnetic baseplate, which should remain attached to the skull.
  c. Attach the baseplate cover to prevent dust on the lens.
  d. Release the mouse and return it to its home cage.

***Note:*** At this point, mice are in principle ready for recording.

### Habituation for EEG/EMG recording and Ca^2+^ imaging during sleep

**Timing: ∼6 h per day for at least 3 days per mouse**

Habituate mice to the miniscope and EEG/EMG cables.

**Figure 9:**
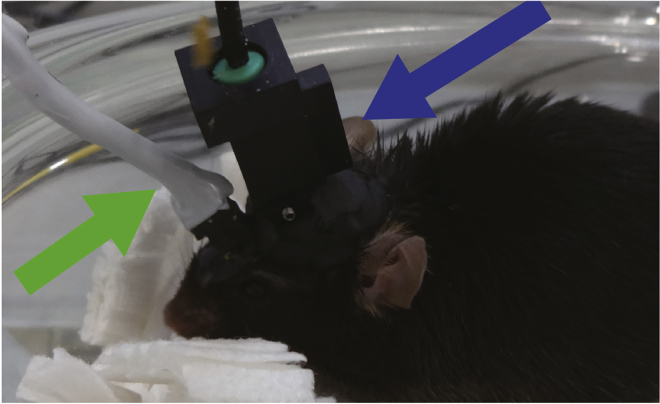
Dummy miniscope and dummy EEG/EMG cables attached to the mouse The dummy miniscope and the dummy EEG/EMG cables are indicated by a blue and a green arrow, respectively.

15. Attach the dummy miniscope and dummy EEG/EMG cables to the mouse.
  a. Hold the mouse head on both sides of the implant (Methods Video S6, 00:00–00:23).
    **CRITICAL:** The mouse will stop moving if its eyes are covered (Methods Video S6). Avoid applying too much force to the implant, which could cause its detachment later.
    Methods Video S6. Connecting the miniscope and EEG/EMG cables to the mouse, refer to steps 15–16
  b. Remove the baseplate cover and attach the dummy miniscope and dummy EEG/EMG cables (Figure 9, Methods Video S6, 00:24–01:39).
    ***Note:*** Mice might initially show difficulties moving around with the miniscope and EEG/EMG cables. We find that a minimum of 9 days of habituation is necessary for mice to freely move. As the goal is only to habituate mice, it is not necessary to use the real recording system at this point.

16. Place the mouse inside the sleeping chamber for ∼6 h (Methods Video S6, 01:40–02:02).

***Note:*** Periodically visually confirm whether the mouse sleeping. Mice should be able to fall asleep more quickly after each habituation session.

17. Finish the habituation protocol.
  a. Remove the mouse from the sleeping chamber.
  b. Detach the dummy materials and attach the baseplate cover.
  c. Return the mouse to its home cage.
18. Repeat the habituation protocol on the following day until the mouse is able to sleep and freely move around inside the sleeping chamber.

### EEG/EMG recording and Ca^2+^ imaging during sleep: day 1, baseline recording

**Timing: 3–6 h per mouse depending on desired imaging period**

***Note:*** For illustrative purposes, we present a protocol for cued fear conditioning as previously described (Kumar et al., 2020).

Record EEG/EMG and Ca^2+^ transients during sleep.

19. Prepare the mouse for EEG/EMG recording and Ca^2+^ imaging.
  a. Start the acquisition software and set the imaging parameters.
    ***Note:*** For consistency, use the same imaging settings/parameters for all mice. For lengthy imaging sessions, we recommend setting the acquisition rate at ∼5 Hz to reduce the file size. For 5 Hz recordings, we find that an LED intensity of 10%–30% of its maximum power for 4 h does not oversaturate any detected pixel. We usually do not observe cell death resulting from phototoxicity during lengthy imaging sessions, but a small degree of photobleaching is inevitable. As the signal-to-noise ratio of GCaMP3 is not as good as that for newer-generation Ca^2+^ sensors, we recommend leaving gain at 1 to reduce imaging noise.
  b. Secure the miniscope and EEG/EMG cables above the recording chamber.
    **CRITICAL:** To avoid damage to the cables, ensure that they do not hang close to the mouse, which can bite and damage them. At the same time, there should be sufficient slack on the cables to avoid movement-induced cable twisting and strain.
    ***Note:*** We placed the EEG/EMG and miniscope cable close to each other so that their rotation axes are similar. During our recording period (maximum ∼4 h), we did not experience substantial tangling of the EEG/EMG and miniscope cables, presumably because we typically perform experiments during the early half of daytime, when mice are often sleeping. If tangling occurs, we recommend using a slip ring for the miniscope with a hole to accommodate the EEG/EMG cable passing through it (or vice versa).
  c. Remove the baseplate cover and attach the miniscope and EEG/EMG cables.

20. Start EEG/EMG recording and Ca^2+^ imaging simultaneously.

**CRITICAL:** Periodically monitor the state of the mouse and cables to ensure that that mouse is behaving naturally and there is no twisting of the cables.

***Note:*** We recommend closely monitoring for signs of sleep apnea. This can generally be achieved by monitoring for fragmented sleep during an NREM sleep episode.

21. Finish the recordings.
  a. Stop the recordings.
  b. Detach both the miniscope and EEG/EMG cables and reattach the dummy miniscope and EEG cable.

### EEG/EMG recording and Ca^2+^ imaging during sleep: day 2, fear conditioning

**Timing: 3–6 h per mouse depending on the research aim**

Fear condition mice with tones and foot shocks.

22. Prepare the mouse for the fear conditioning chamber.
  a. Remove the baseplate cover and attach the miniscope.
  b. Start the Ca^2+^ imaging acquisition software and set the imaging parameters.
  c. Arrange the cables above the conditioning chamber by taking the same precautions as those used for the sleeping chamber.

***Note:*** Use the dummy miniscope if the actual recoding equipment is not needed during conditioning.

23. Fear condition the mouse.
  a. Place the mouse inside the conditioning chamber.
  b. Perform Ca^2+^ imaging for 10 min during the pre-conditioning period (i.e., pre-shock recording).
  c. Stop the imaging and quickly detach the miniscope and reattach the dummy miniscope (<1 min) to avoid a change in either the shock condition or the field of view (due to hitting the miniscope against the wall during shock).
  d. Perform cued fear conditioning (Methods Video S7).
    Methods Video S7. Fear conditioning with the miniscope attached to the head, refer to step 23d
  e. Quickly reattach the miniscope and perform 5 min of Ca^2+^ imaging (i.e., post-shock recording).
  f. Finish the recording and return the mouse to the sleeping chamber.
  g. Connect the EEG/EMG cable and start EEG/EMG recording and Ca^2+^ imaging simultaneously.

24. Record EEG/EMG activity and Ca^2+^ transients.
  a. Transfer the sleeping chamber to the sleep recording room.
    ***Note:*** If necessary, it is possible to record the activity of neurons during the foot shocks of fear conditioning (Grewe et al. 2017). In this case, we recommend covering the interior walls of the conditioning chamber with a shock-absorbing material to prevent damage to the miniscope if the mouse hits the walls when reacting to the foot shocks.
    ***Note:*** Avoid detaching the miniscope and cables from the head of the mouse when transferring it to the sleeping chamber to avoid a change in the field of view. If necessary, disconnect the cables from the computer port and reconnect them after transferring the mouse.
    ***Note:*** If the mouse is not properly habituated to the experimental manipulation, it is possible that some procedures (e.g., plugging and unplugging the cables/miniscope) could stress the mouse and affect its freezing response. Therefore, we suggest confirming whether freezing is specifically observed in the shocked context. For a no-learning control group, a no shock or immediate shock protocol can be considered.
  b. Start the EEG/EMG recording and Ca^2+^ imaging simultaneously.

25. Finish the recording as done on the previous day.

### EEG/EMG recording and Ca^2+^ imaging: day 3, fear conditioning test

**Timing: 5–10 min per mouse**

Confirm the establishment of a fear memory.

26. Prepare the mouse for the fear conditioning chamber as done on the previous day.

***Note:*** Use the dummy miniscope if the actual recoding equipment is not needed during conditioning.

27. Confirm the establishment of a fear memory.
  a. Start EEG/EMG recording and Ca^2+^ imaging software.
  b. Place the mouse inside the conditioning chamber.
  c. Start the EEG/EMG recording and Ca^2+^ imaging simultaneously.
  d. Leave the mouse inside the chamber for 5–10 min.

***Note:*** Attaching the miniscope during the test tends to reduce the overall amount of freezing (Kumar et al., 2020). To avoid confounding effects, we habituated mice to the weight of the miniscope using a dummy miniscope and cables. A similar approach is widely adopted in tetrode experiments. We found that although mice tend to show low freezing behavior when the miniscope is attached, they freeze only in the learning context (Kumar et al., 2020). If the freezing duration is still too short, longer habituation with the dummy microscope or counterbalancing the microscope should be considered. Alternatively, a slip ring could be used.

28. Finish the recording as done on the previous day.

### Anatomical confirmation of the recorded signals

**Timing: dependent on the desired histological method**

Verify the position of the implanted GRIN lens post-mortem.

29. Obtain a brain sample
  a. Euthanize the mouse according to institutional guidelines.
  b. Transcardially perfuse the mouse with cold 4% paraformaldehyde (PFA).
  c. Remove the brain and place it inside a tube with 4% PFA at 4°C for 24 h.

**Pause point:** The brain can be stored at 4°C for several days. In this case, exchange the PFA for phosphate-buffered saline (PBS) after 24 h and protect the tube from light, but expect a natural decay of the fluorescence signal over time. Long exposure to PFA may interfere with immunohistochemical detection of antigens, some of which are critical for adult neurogenesis research, such as doublecortin (Moreno-Jimenez et al., 2019).

***Optional:*** A peristaltic pump can be used for perfusion. Replace the 4% PFA with 20%–30% sucrose solution if a cryostat will be used to slice the brain. After the brain sinks, remove it from the tube and cryopreserve it in an OCT mounting medium block at −80°C.

30. Slice the brain.
  a. Slice the brain using a cryostat or vibratome at 50 μm thickness and collect the slices in plastic wells or dishes with desired buffer.

***Note:*** We use 60% glycerol/PBS solution for long-term storage of brain slices in a freezer. In case slices must be kept at 4°C, we recommend adding 0.1% sodium azide to the buffer to prevent bacterial contamination.

**Figure 10:**
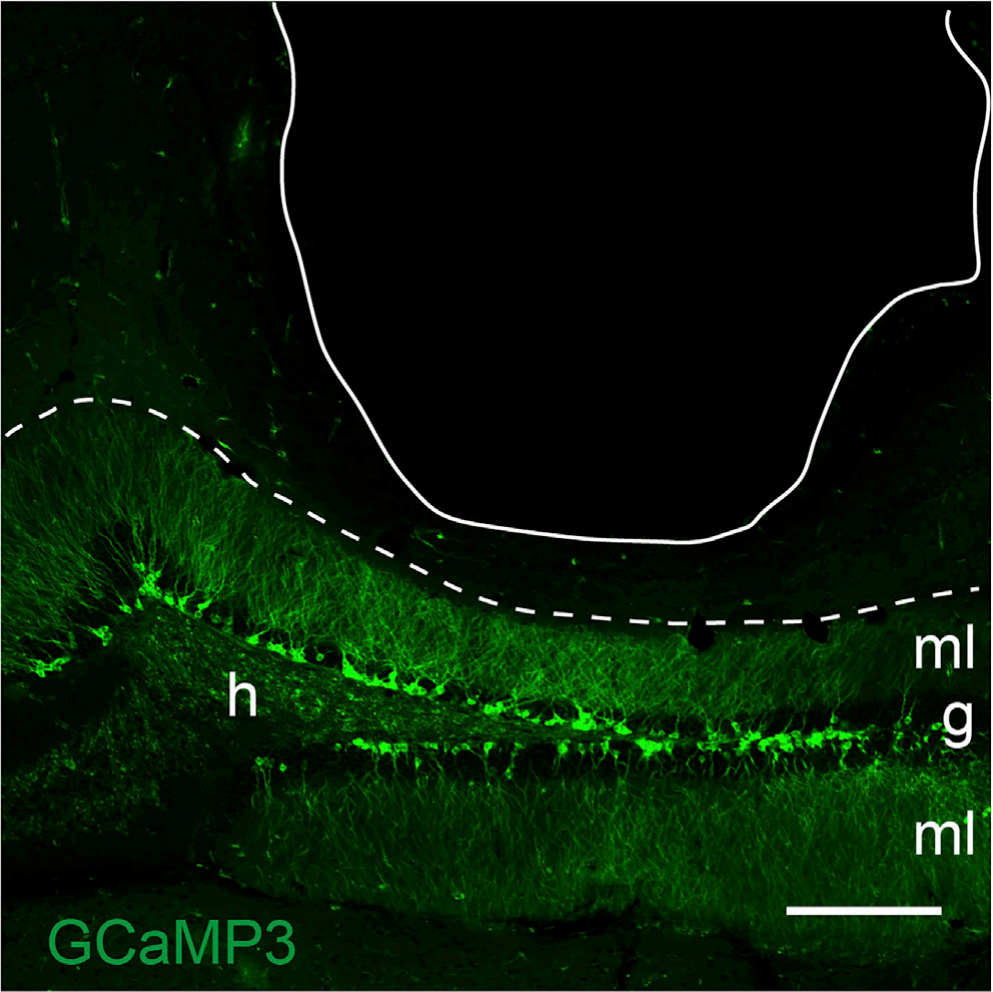
Histological confirmation of GRIN lens position and GCaMP3 signal The letters g, h, and ml indicate the granule cell layer, hilus, and molecular layer of the DG, respectively. The white line indicates the position of the lens, and the white dotted line indicates the border between the DG and stratum lacunosum moleculare. The expression of GCaMP3 is shown in green. Scale bar, 100 μm.

***Note:*** GCaMP3 fluorescence is usually sufficiently bright to be detected without immunohistochemical staining. If it is not possible to observe the fluorescence signal due to a particular reason (e.g., acetone pretreatment), a simple antibody stain targeting the GFP molecule is sufficient to confirm GCaMP3 expression. We found that using Malinol mounting medium is an economical option for rapid confirmation of the signal.

31. Confirm the location of the implanted lens and GCaMP3 fluorescence signal.
  a. Mount the brain slices on microscope slides using fluorescence-preserving medium.
  b. Image the slides with a fluorescence microscope (Figure 10).

## Expected outcomes

Successful completion of this method allows the detection of ABN Ca^2+^ transients in freely moving mice. A recording session configured with the parameters described here will produce a raw recording file with a size of ∼1,327 kb/frame. A representative recording video of Ca^2+^ transients is provided in our previous article (Kumar et al., 2020).

## Quantification and statistical analysis

Here, we present steps for processing raw video files into Ca^2+^ fluorescence time series. We use Mosaic software for preprocessing and motion correction and the MATLAB implementation of the constrained non-negative matrix factorization (CNMF)-E algorithm (Zhou et al., 2018) and custom scripts to optimize extraction of ABN Ca^2+^ transients (available through the GitHub link below).

***Note:*** We recommend the following system requirements: ≥128 GB RAM; MATLAB 2020 or above; most recent Intel Core i7 processor; 1 TB SSD.

1. Decompress the raw files using the Inscopix image decompressor.

***Note:*** We recommend downsampling the video by a factor of 4 during decompression to save disc space and reduce loading times. Decompressing video files into .hdf5 format substantially improves loading times in Mosaic and MATLAB.

2. Preprocess the video file according to the standard Mosaic workflow (Methods Video S8, 00:00–00:22).
  a. Import the video file into Mosaic software.
  b. Preprocess the file in Mosaic. Select “fix defective pixels” and “fix isolated dropped frames.”
  Methods Video S8. Analyzing Ca^2+^ imaging data, refer to quantification and statistical analysis steps 2–5

***Note:*** Spatial downsampling is not necessary if already done during decompression.

3. Perform motion correction according to the standard Mosaic workflow (Methods Video S8, 00:22–01:25).
  a. Save the file in “.h5” format.

***Note:*** Preprocessing and motion correction can be performed using any version of Inscopix Data Processing Software (https://www.youtube.com/watch?v=YF4IlcotTrM&feature=emb_logo). Motion correction can be performed using CaImAn (Giovannucci et al., 2019) or any other software while considering the specific characteristics of ABN activity as described below.

***Note:*** Ideally, perform optimal motion correction by choosing a reference point in the field of view where most ABNs are located with clear spatial markers (i.e., blood vessels). This is important because movements of the brain occur in three dimensions and might be non-rigid. Thus, perfect motion correction in the entire field of view is not possible in most cases. Instead, choose reference regions that minimize motion in the region where ABNs are located and consider cropping out unnecessary portions of the video, which will reduce the number of false positives and decrease processing time. Moreover, some regions in the field of view may move asynchronously. This can occur when tissue or blood clots get stuck between the lens and focal plane, which creates different planes of movement in the image. Try to avoid analyzing regions that contain desynchronized movement.

***Note:*** Detecting ABN activity by the naked eye can be challenging. This is because ABNs are low in number (∼5–25 ABNs per field of view) and very sparsely active (∼1 transient/min). Moreover, the GCaMP3 signal is weaker than newer-generation GCaMPs. To improve the identification of regions with active ABNs, adjust the contrast to between 40%–95% of the maximum observed signal. An estimation of neuronal location can be obtained in the motion correction panel when you check “subtract spatial mean,” “invert image,” and “apply spatial mean,” which makes ABNs visible as black circular shapes in the field of view.

**CRITICAL:** After performing motion correction, check that no major signs of motion are visually detected. A single run of the motion correction algorithm is usually not sufficient to solve all problems. Instead, try using different combinations of reference region and motion correction type (e.g., translation, rotation). We recommend first using “translation only” as the motion correction type. If there is still motion that was not corrected, check whether this is due to rotations or expansions in the field of view and change the motion correction type accordingly.

***Note:*** For information on how to perform motion correction when tracking the same ABNs through different recording sessions, see the “tracking the same neurons” section in our previous article (Kumar et al., 2020).

4. Extract Ca^2+^ transients in MATLAB using CNMF-E (Methods Video S8, 1:25–6:25).
  a. Set up a folder with the “.h5” video files to analyze.
  b. Add the folder with the CNMF-E scripts provided in this article to the MATLAB path.
  c. Open the file “ABNs_PV.m.”
  d. Input the path of the folder with the video files in line 2.
  e. Input the frame rate in line 46.
  f. Run the code.

***Note:*** For short video sessions, run “demo_large_data_1p.m” instead of “ABNs_PV.m.”

5. Perform the following quality controls:
  a. Run “neuron.viewNeurons([], neuron.C_raw);” to visually inspect each neuron.
    ***Note:*** Delete extracted components with temporal traces or spatial shapes that do not correspond to real neurons. If you must delete too many neurons, consider repeating the analysis with higher peak-to-noise ratio (PNR) or local correlation (CORR) thresholds.
  b. Run “neuron.show_contours(0.6, [], neuron.PNR.∗neuron.Cn, 1)” to see the contours of the extracted components over the PNR image multiplied by the CORR image.
    ***Note:*** Enlarged non-circular regions usually reflect motion artifacts. If contours are not drawn over several neuron-like regions, this suggests that the PNR or CORR threshold were set too high. If contours are drawn over several non-neuron-like regions, this suggests that the PNR or CORR threshold were set too low.
  c. Run “implay(cat(2,mat2gray(neuron.PNR_all),mat2gray(neuron.Cn_all)),5);” to see the PNR and CORR images from consecutive temporal segments of 1,000 frames.
  d. Check for signs of motion artifacts and the presence of a visual representation of the mean activity of ABNs across different times.
  e. Run stackedplot(neuron.C_raw); to see several Ca^2+^ transients at the same time.

***Note:*** Correlated ensemble activity is common in ABNs; however, perfectly overlapping rising dynamics with square-like shapes may indicate motion artifacts. Check the video in the specific frames to corroborate whether this is real ensemble activity (example in Methods Video S8).

**Pause point:** To extract Ca^2+^ transients, we use CNMF-E (Zhou et al., 2018) with some modifications. We recommend first reading the original CNMF-E article to understand the main concepts behind the algorithm. We choose CNMF-E because it is particularly good at extracting noisy signals, and the code is easily adapted to a wide range of experimental needs. We made some minor modifications to the original code that are necessary for lengthy recordings (>10 min), which is available in GitHub link below. For shorter recordings, we recommend using the original code.

**Figure 11:**
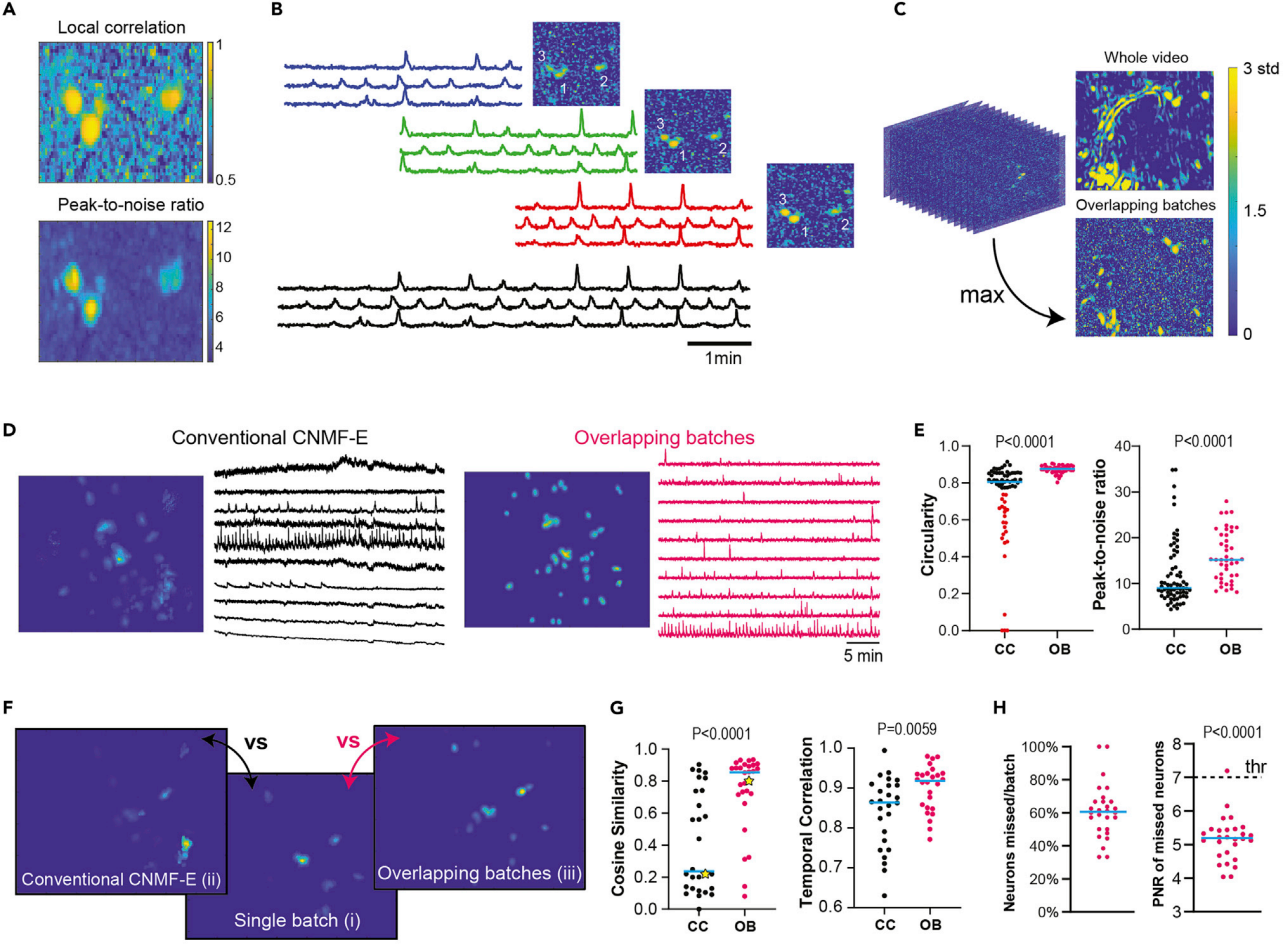
Analysis of Ca^2+^ imaging data Overlapping batch analysis. (A) Local correlation (CORR) and peak-to-noise ratio (PNR) image of three neurons in a recording file. (B) Ca^2+^ transients detected and CORR for three neurons in three consecutive batches. Black trace shows the final estimated signal for the entire recording. (C) Normalized CORR for a 3-h video obtained by analyzing the entire video sequence versus the maximum projection of the CORR images obtained by overlapping batch implementation. Note that CORR of the entire video sequence includes many highly correlated non-circular shapes that are unlikely to represent real ABNs. Analysis of the entire video will introduce several false positives and noisier estimation of Ca^2+^ transients in this case. (D) Spatial and temporal features of Ca^2+^ transients extracted by conventional CNMF-E (CC) and overlapping batches (OB) methods. The two transients were randomly chosen from those extracted by CC or OB methods. (E) Circularities (isoperimetric quotient) of spatial components and PNR of temporal traces. (F) Example of spatial components extracted from an individual batch (i), the OB method (ii), or the CC method (ii) in the same time period. Because there are more neurons in the entire recording period than in an individual batch, spatial components were weighted by average activity during the period. (G) Left: Cosine similarity of spatial components for the individual batch versus CC method and individual batch versus OB method. The star symbol in the figure corresponds to the spatial components shown in (F). Right: Temporal correlation of Ca^2+^ transients of common neurons for the individual batch versus CC method and individual batch versus OB method. A neuron extracted by the CC or OB method was considered to be the same as that in an individual batch if its spatial component had a cosine similarity >0.8. If more than two pairs of neurons satisfied this condition, the pair with the higher temporal correlation was used for calculations. (H) Left: percentage of active neurons not detected in an individual batch. Right: PNR of missed neurons. Data were analyzed by Mann-Whitney tests (E, G) and one-sample t tests (H).

The parameters that mainly affect CNMF-E results are the minimum local correlation of a pixel and its neighbors (Figure 11A, left; noted as “min_corr”) and the minimum PNR of a pixel (Figure 11A, right; noted as “min_pnr”). These parameters are thresholds used to obtain the first estimation of the spatial and temporal components that will be used to initialize the CNMF algorithm. For sparse data, such as that from ABNs, total video length may affect the estimation of CORR. This happens because in one-photon imaging, pixels associated with a neuron are highly correlated only when that neuron is active. Given that ABNs are usually inactive, increasing the video length may be translated into a poor CORR, especially for files that are several hours long.

To solve this problem, we segment the video in short overlapping batches of 1,000 frames, which allows us to estimate the spatial component of ABNs from discrete temporal windows in which they are highly active. This is depicted in Figure 11B, in which the CORR and Ca^2+^ transients of three close ABNs are shown for three consecutive batches. Note that ABNs display prominent Ca^2+^ transients that translate into a higher CORR, particularly in the third batch. Because the spatial component of each neuron is shared across batches, only this batch is necessary to initialize those ABNs, even if other batches have CORR comparable to the background level. Having obtained a good initial estimation of spatial components, the CNMF algorithm can extract temporal traces from other batches, even if its CORR or PNR is below the defined threshold. An overlapping batch approach produces a cleaner estimation of CORR, in contrast to the analysis of the entire video sequence (Figure 11C).

The multi-batch algorithm is implemented in the original CNMF-E article (Zhou et al., 2018); however, we find that in some cases, artifacts are introduced in the concatenation point between batches. Thus, we implement a multi-batch algorithm using overlapping batches. We also include some utility functions to correct for variations in baseline noise between batches and some minor bugs producing empty matrices given the sparse activity of ABNs. Our code contains optimized parameters that can extract Ca^2+^ transients from ABNs in most cases. To illustrate the differences between the overlapping batches (OB) method and the conventional CNMF-E (CC) method (i.e., running CNMF-E on the whole video sequence), we compared the spatial and temporal components extracted by both methods (Figure 11D). True-positive neurons are expected to display circular shapes and Ca^2+^ transients distinguishable from noise. Neurons extracted by the OB method display higher circularity and PNR than those extracted by the CC method (Figure 11E). Indeed, several neurons extracted by the CC method display spatial components that largely differ from a circular shape. Considering those spatial components with circularities five standard deviations below those extracted by the OB method, we estimated that 34% of components extracted by the CC method are false positives (Figure 11E, left, red dots). Next, we examined how the final temporal and spatial components of the transients extracted by the OB or CC method (analysis of >30,000 frames) differ from those extracted from the analysis of an individual batch (analysis of ∼1,000 frames) (Figure 11F). Components extracted by the OB method were spatially and temporally more similar to those extracted from an individual batch (Figure 11G). We estimate that, on average, 61% of neurons remain undetected in individual batch analysis compared with the OB method (Figure 11H, left) because they display PNR significantly below the detection threshold. These results indicate that the OB method minimizes the number of false positives without compromising true-positive detection.

6. To track ABNs across different sessions, motion correct each session as previously described (Ghandour et al., 2019). In Mosaic software, this is done as follows:
  a. Motion correct each session independently.
  b. Extract one frame from one session to use as a reference for other sessions.
  c. Motion correct each session relative to the extracted frame.
    ***Note:*** Sometimes using different reference frames from different sessions provides better results. Consider the points mentioned in “Quantification and statistical analysis: step 3.”
  d. Concatenate the recording sessions, ensuring that sessions are correctly aligned.

**Pause point:** Tracing ABNs across several days using tracking algorithms (Sheintuch et al., 2017) may not be possible given the small number of ABNs. However, we were able to track ABNs across different recording sessions within the same day by concatenating different sessions into one file. Spatial markers, such as blood vessels or ABNs with persistent activity, can be used to align different sessions.

Analyzing sessions independently and then identifying the same ABNs based on their footprints assumes that all ABNs can be detected in each recording session. However, this is not always the case, especially considering the sparse activity of ABNs and the low signal-to-noise ratio of GCaMP3. This issue is illustrated in Figure 11B; note that in the first batch, three ABNs are active with CORR comparable to the background local correlation. If lower CORR and PNR thresholds are used to detect these ABNs, many false-positive neurons would be included in the analysis, further hindering the tracking of ABNs. Instead, we initialize these ABNs in a specific period in which they show high activity (e.g., third batch in Figure 11B). The CNMF algorithm is then used to extract Ca^2+^ transients from other batches with a lower signal-to-noise ratio. This is particularly important when tracking the same ABNs because some may be more active in some recording sessions than in others and, hence, be wrongly labeled as inactive if these sessions are analyzed independently.

7. After extracting the raster plot of ABN activity, we further analyzed data for each sleep stage in 10-s bins using Sleep Sign software (example raw data are available in Kumar et al., 2020). Alternatively, several other types of software are available for sleep analysis (e.g., Sleep, Combrisson et al., 2017). More details of sleep recording and analysis are available in Oishi et al. (2016).

## Limitations

Although GCaMP imaging and miniscope technologies fuel new discoveries of how neuron activity generates specific behaviors in freely moving animals, their limitations are well known.

One limitation is related to the resolution of the z-axis. The miniscope used in our study (Kumar et al., 2020) excites fluorescence molecules without focusing or scanning the light; it does not have a pinhole. Therefore, all fluorescence molecule hundreds of micrometers below the miniscope can be excited and detected at the same time. This can allow researchers to obtain a larger number of ABN signals. However, it is possible that a detected Ca^2+^ transient can stem from two ABNs aligned in the z-axis.

Another limitation is that the experimental preparation for DG imaging in freely moving mice results in a partial lesion of the ipsilateral CA1 area when implanting the GRIN lens. Less invasive techniques could be used in the future to prevent possible circuit reorganization induced by surgery, as previously recommended (Hainmueller and Bartos, 2018; Kirschen et al., 2017; Pilz et al., 2016).

Finally, the developing nature of ABNs imposes a time constraint on the experimental schedule. As the activity and behavioral significance of ABNs change as they transition from immature to mature granule cells (e.g., Kumar et al., 2020), it can be challenging to use the same mice in multiple behavioral tasks and ensure that the recorded ABNs have the same physiological properties across the task periods.

A final limitation is the overall cost of the necessary equipment. Using the described hardware makes the experiment faster and easier to perform, but it costs more than $150,000 USD in total. Fortunately, more budget-friendly options are available. A similar miniscope can be built by a researcher using the open-source miniscope platform (UCLA Miniscope). In addition, simpler over-the-counter computers can be used for the experiments, but this would likely increase the data processing time.

## Troubleshooting

### Problem 1

GCaMP3-labeled cells are rare or absent (steps 1, 2, and 31).

### Potential solution

The presence of a small number of GCaMP3-labeled neurons can be due to inefficient Cre induction/recombination by tamoxifen administration. The dose-dependency of GCaMP3 expression should be confirmed in each experimental setting. Always prepare fresh tamoxifen solution, protecting it from light. Ensure that tamoxifen is completely dissolved to achieve a sufficient dose. Also, when injecting, leave the needle tip in the intraperitoneal space for a few seconds to avoid reflux of tamoxifen. The injected dose can be systematically increased to determine whether this solves the problem.

When using different versions of GCaMP, confirm immunohistologically that Cre recombinase is expressed and that there is no cell death induced by cytotoxicity of Ca^2+^ sensors in ABNs.

### Problem 2

The lens is incorrectly positioned (steps 4–9).

### Potential solution

This problem can occur due to variations in different stereotaxic equipment or unintended changes in lens position during implantation. Confirm that the lens is oriented precisely within the hole and re-evaluate the coordinates for each experimental setting.

Implanting the lens into the CA1 generates resistance, which can force the lens out of the brain. Only release the lens from the stereotaxic micromanipulator after the glue/dental cement cures; otherwise, the position of the lens may shift when released from the grip.

### Problem 3

The implant detaches from the mouse (steps 6–28).

### Potential solution

Implant detachment can occur when it is not well secured to the skull. When applying several layers of different adhesives, ensure that each layer completely cures before applying the next one, as mixing them could compromise their stability. In some cases, standard black cement may compromise the longevity of the implant. One possibility for this case is to use regular light dental cement and cover it with black paint or nail polish to prevent ambient light-derived artifacts. Infection and tissue inflammation can also decrease the adhesive strength of the glue/dental cement; ensure adequate prophylactic practices during surgery and postoperative recovery to prevent this problem. In addition, do not immobilize mice by holding the baseplate, as physical stress at the implant can cause its detachment.

### Problem 4

Excessive motion artifacts are present during data acquisition (steps 12–14, 19, 24, and 27).

### Potential solution

Ensure that the baseplate is fixed properly on the skull and that the miniscope is correctly attached to the baseplate and tightly secured in place by its screw. Also, low frame rates may increase the amount of motion blur, which can impair performance of the motion correction algorithm.

### Problem 5

Fluorescence signals do not show dynamic changes in intensity (steps 13, 20, 24, 27).

### Potential solution

“False” fluorescence signals are usually derived from autofluorescent materials (e.g., damaged tissue or blood cells below the lens) and not from GCaMP3. To prevent these, ensure that the cortex above the target coordinates is completely aspirated and that there is no residual bleeding before implanting the lens. As lowering the lens too quickly into the brain can damage the tissue, ensure that the lens is implanted slowly and allow the tissue to settle after each 150-μm step. If necessary, retract the lens ±25 μm before each step to further alleviate pressure and prevent unnecessary damage.

### Problem 6

Data processing is extremely slow or impossible (Quantification and statistical analysis steps).

### Potential solution

As sleep recordings can be >3 h long, the final compressed file can be >200 GB. To be processed, the file will need to be decompressed and spatially down-sampled (4×), resulting in a ∼216 kb/frame file. However, because some MATLAB scripts require double precision, we recommend that the PC has at least four times more RAM than the down-sampled file size. When it is not possible to reduce the size of the data file by temporal and/or spatial binning, the full-length recording can be split into two shorter files. The initial processing steps can be performed separately for each file, which can later be concatenated before detecting active ABNs using batch implementation and disabling parallel computing (although this can take several hours).

### Problem 7

Few or no ABNs are present in the imaging file (Quantification and statistical analysis step 4).

### Potential solution

If there are no problems with GCaMP3 expression or lens implantation coordinates, this problem can be solved by changing parameters in the CNMF-E script. Start by modifying the minimum CORR and PNR thresholds. In this case, visual inspection of cell shape and temporal dynamics of Ca^2+^ transients become more important, as the rate of false cell detection increases with less stringent parameters.

### Problem 8

MATLAB code crashes during analysis (Quantification and statistical analysis steps 4–6).

### Potential solution

The code (original or provided) is not bug-proof. In case of an error, read the error message in the MATLAB command window. If no ABN is detected, the code will crash. Visually confirm whether you can detect Ca^2+^ activity. If active ABNs can be visually detected in the raw file, consider using a lower PNR or CORR threshold. Also, monitor your RAM usage when running the code. Consider upgrading your hardware or reducing the recording duration.

## Resource availability

### Lead contact

Further information and requests for resources and reagents should be directed to and will be fulfilled by the Lead Contact, Masanori Sakaguchi (sakaguchi.masa.fp@alumni.tsukuba.ac.jp).

### Materials availability

We did not generate any unique/stable reagents in this study.

### Data and code availability

Data and code underlying the results described in this manuscript are available at:

https://github.com/vergaloy/ABNs-Calcium-extraction

Also, the raw data from the related article (Kumar et al., 2020) are available at:

https://data.mendeley.com/datasets/gfbdv5kfrz/1
